# Identification of Quantitative Disease Resistance Loci Toward Four *Pythium* Species in Soybean

**DOI:** 10.3389/fpls.2021.644746

**Published:** 2021-03-30

**Authors:** Elizabeth M. Clevinger, Ruslan Biyashev, Elizabeth Lerch-Olson, Haipeng Yu, Charles Quigley, Qijian Song, Anne E. Dorrance, Alison E. Robertson, M. A. Saghai Maroof

**Affiliations:** ^1^School of Plant and Environmental Sciences, Virginia Tech, Blacksburg, VA, United States; ^2^Department of Plant Pathology and Microbiology, Iowa State University, Ames, IA, United States; ^3^Department of Animal Science, Iowa State University, Ames, IA, United States; ^4^Soybean Genomics and Improvement Laboratory, Agricultural Research Service, United States Department of Agriculture, Beltsville, MD, United States; ^5^Center for Applied Plant Sciences and Soybean Research, Department of Plant Pathology, Ohio State Sustainability Institute, The Ohio State University, Wooster, OH, United States

**Keywords:** *Pythium*, quantitative disease resistant loci, soybean, resistance, recombinant inbred line population

## Abstract

In this study, four recombinant inbred line (RIL) soybean populations were screened for their response to infection by *Pythium sylvaticum*, *Pythium irregulare*, *Pythium oopapillum*, and *Pythium torulosum.* The parents, PI 424237A, PI 424237B, PI 408097, and PI 408029, had higher levels of resistance to these species in a preliminary screening and were crossed with “Williams,” a susceptible cultivar. A modified seed rot assay was used to evaluate RIL populations for their response to specific *Pythium* species selected for a particular population based on preliminary screenings. Over 2500 single-nucleotide polymorphism (SNP) markers were used to construct chromosomal maps to identify regions associated with resistance to *Pythium* species. Several minor and large effect quantitative disease resistance loci (QDRL) were identified including one large effect QDRL on chromosome 8 in the population of PI 408097 × Williams. It was identified by two different disease reaction traits in *P. sylvaticum*, *P. irregulare*, and *P. torulosum*. Another large effect QDRL was identified on chromosome 6 in the population of PI 408029 × Williams, and conferred resistance to *P. sylvaticum* and *P. irregulare*. These large effect QDRL will contribute toward the development of improved soybean cultivars with higher levels of resistance to these common soil-borne pathogens.

## Introduction

Soybean [*Glycine max* (L.) Merr.] is a major oil and protein crop grown worldwide. In the United States in 2017, soybean was planted on more than 40 million production acres with a value of $41 billion (American Soybean Association, 2017). Seed and seedling diseases of soybean have been reported in all soybean growing regions of the United States and can be a constraint to production (Wrather and Koenning, 2006). Reduced stand, seed rot, and seedling damping-off are symptoms of seedling diseases. Moreover, if infected plants survive, they may be less vigorous with reduced yield (Broders et al., 2007). From 2010 to 2014, seedling diseases were among the top 10 most yield-limiting diseases of soybean in both the northern and southern United States (Allen et al., 2017).

There has been considerable focus on the seedling diseases caused by oomycete pathogens, such as *Phytophthora sojae* and numerous *Pythium* species (Broders et al., 2007, 2009; Jiang et al., 2012; Zitnick-Anderson and Nelson, 2015; Rojas et al., 2017). In the most recent survey, species of *Pythium* were widespread in 11 major soybean-producing states in the United States and Ontario, Canada. Of the more than 79 species of *Pythium* recovered from symptomatic soybean seedlings, more than 30 were capable of causing seedling disease on the soybean cultivar “Sloan” (Rojas et al., 2017). In North Dakota, 26 species of *Pythium* were recovered over a 2-year period of sampling (Zitnick-Anderson and Nelson, 2015). In the North Central region of the United States, the most prevalent species of *Pythium* recovered from diseased soybean seedlings was *Pythium sylvaticum* (Broders et al., 2007; Rojas et al., 2017). Other species that frequently occurred included *Pythium oopapillum*, *Pythium heterothallicum*, *Pythium ultimum var ultimum*, *Pythium irregulare, Pythium torulosum*, and *Pythium lutarium*. Although a single species of *Pythium* is capable of causing disease, several species can be isolated from the same plant (Rizvi and Yang, 1996; Dorrance et al., 2004; Broders et al., 2007; Zitnick-Anderson and Nelson, 2015). *Pythium* spp. have a wide host range including corn (*Zea mays* L.) (Zhang and Yang, 2000), wheat (*Triticum aestivum* L.) (Ingram and Cook, 1990), and cotton (*Gossypium hirsutum* L.) (Wang and Davis, 1997), crops that are commonly grown in rotation with soybean in different areas of the United States.

Soybean production practices in areas of the Midwest intensify the risk of pre-emergence damping-off. Recently, there has been a trend for earlier planting dates, when soils are cooler. Emergence is delayed at low temperatures; thus, seeds are exposed to pathogens in the soil for longer, thereby increasing the risk of seedling disease (Serrano and Robertson, 2018). Although seedling disease is associated with cool and wet conditions, it can occur over a range of temperatures as different species may have different temperature requirements for pathogenicity (Matthiesen et al., 2016; Rojas et al., 2017). This variation for optimal temperatures suggests that the predominant species causing disease in a field will depend on the temperature of the soil prior to and at planting. You et al. (2017) found in a modeling study of a legume that a complex relationship exists between factors such as temperature, soil and moisture, and *Pythium* damping-off and root rot. Thus, even if planting is delayed until soils warm up, seedling disease may still be a risk. Furthermore, reduced tillage practices are common across the Midwest which allow for a build-up of inoculum in the top layers of soil (Broders et al., 2007).

Seed and seedling rot caused by *Pythium* species are managed by fungicide seed treatments (Bradley, 2008; Radmer et al., 2017). Metalaxyl, mefenoxam, and ethaboxam are fungicides used to control *Pythium* species; however, some less sensitive isolates have been reported (Dorrance et al., 2004; Broders et al., 2007; Radmer et al., 2017). Most fungicide seed treatments only last for 10–14 days and cannot efficiently protect developing roots (Paulsrud et al., 2005). Therefore, it is important to have additional management strategies in place.

Two types of host resistance have been identified for *Pythium* species in soybean, monogenic and partial resistance. Monogenic resistance to *Pythium aphanidermatum* was identified in an F_2:4_ population of “Archer” × “Hutcheson” (Rosso et al., 2008). Recently, several quantitative disease resistance loci (QDRL) for resistance to *P. irregulare* (Ellis et al., 2013; Stasko et al., 2016; Lin et al., 2018; Scott et al., 2019), *P. aphanidermatum* (Urrea et al., 2017), *P. ultimum* var *sporangiiferum* (Scott et al., 2019), *P. sylvaticum* (Lin et al., 2020), and *P. ultimum* var. *ultimum* (Rod et al., 2018; Klepadlo et al., 2019; Scott et al., 2019) have been identified using biparental soybean populations. Overall, only minor QDRL have been identified and have explained 4.5–17.8% of the phenotypic variation and are located on most chromosomes within the soybean genome.

In this study, four recombinant inbred line (RIL) populations were screened for their response to infection by *P. sylvaticum*, *P. irregulare*, *P*. *oopapillum*, and *P*. *torulosum.* These species of *Pythium* were used based on their prevalence in the north central region of the United States and Ohio (Broders et al., 2007, 2009; Rojas et al., 2017). The parents, PI 424237A, PI 424237B, PI 408097, and PI 408029, had higher levels of resistance to these species in a preliminary screening (Lerch-Olson et al., unpublished). A modified seed rot assay was used to screen the RILs for their response to each species. Several minor and large effect QDRL were identified including one large effect QDRL on chromosome 8, which was detected by three different *Pythium* species and a large effect QDRL on chromosome 6 detected by two different *Pythium* species.

## Materials and Methods

### Plant Material

Genetic materials for this study included four RIL populations (Table 1). Williams × PI 424237A (POP1), an F_8_ RIL population with 137 lines, PI 424237B × Williams (POP2), an F_7_ RIL population with 169 lines, and an F_9_ RIL population of PI 408097 × Williams (POP3) with 307 lines were planted in 2017, at Kentland Farm in Blacksburg, VA, United States. A fourth population, PI 408029 × Williams (POP4), an F_7_ RIL population with 198 lines was planted in 2016, at this same location.

**TABLE 1:** Summary of four populations of soybean assessed for their response to species of *Pythium* in a seed rot assay.

Population	Population size	Generation	Species assayed
Williams × PI 424237A (POP1)	137	F9	*P. sylvaticum*, *P. oopapillum*
PI 424237B × Williams (POP2)	169	F8	*P. torulosum*, *P. oopapillum*
PI 408097 × Williams (POP3)	307	F10	*P. torulosum*, *P. sylvaticum*, *P. irregulare*
PI 408029 × Williams (POP4)	198	F8	*P. sylvaticum*, *P. irregulare*

Young first or second trifoliate leaves of greenhouse-grown plants were collected for DNA extraction. DNA from parental lines and at least 10 bulked plants from each individual RIL was isolated from lyophilized tissues by using the CTAB method as described in Saghai Maroof et al. (1984), with minor modifications. DNA concentration was measured with a DyNA Quanta2000 Fluorometer (Hoefer^®^Scientific, San Francisco, CA, United States).

### Disease Screening

All disease screenings were conducted at Iowa State University in 2018 in a growth room without light (Lerch-Olson et al., 2020). Each of the four populations was evaluated for resistance to two or three *Pythium* species (Table 1) in a modified seed rot assay (Zhang and Yang, 2000; Broders et al., 2007). Briefly, for each species, one *Pythium* isolate was transferred to diluted V8 juice medium with antibiotics (DV8++; 40 mL V8 juice, 0.6 g CaCO_3_, 0.2 g Bacto yeast extract, 1 g sucrose, 0.01 g cholesterol, 20 g Bacto agar, 1 L distilled water, 0.05 g L^–1^ neomycin sulfate, and 0.01 g L^–1^ chloramphenicol) on 9 cm petri plates and then incubated in the dark at room temperature for 4 days. Seeds were surface sterilized in a 1% solution of sodium hypochlorite for 3 min and then rinsed in sterile water for 3 min and dried with sterilized paper towels. Five seeds from each RIL were placed on 4-day-old *Pythium* colonized plates and the plates were incubated in the dark for 7 days at 15°C (*P. oopapillum* and *P. torulosum*) or 23°C (*P. irregulare* and *P. sylvaticum*). The cultivar “Sloan” was used as a susceptible check (Lerch-Olson et al., 2020). Previous work identified “Sloan” as susceptible to multiple *Pythium* species (Broders et al., 2007; Matthiesen et al., 2016). Each plate was considered as one replication and there were four replications within each experiment (20 seeds/run). In each experiment and run, one non-inoculated plate with 10 seeds was included to determine the germination of each RIL. There were two experiments (40 seeds/RIL) for each of the species in a completely randomized design. Seed rot was evaluated using a severity scale (SRS) adapted from Zhang and Yang (2000). The scale was from 0 to 4, where 0 indicated seeds that germinated and had no radicle discoloration; 1 indicated germinated seeds with less than 50% radicle discoloration; 2 indicated germinated seeds with more than 50% radicle discoloration; 3 indicated that the seed germinated but rotted; and 4 indicated rotted seeds that had never germinated. Germination was considered when the length of the radicle was equal to the length of the seed. Data were also taken on the percent of rotted seeds per inoculated plate (ROTS) and the adjusted germination (AGERM). AGERM was calculated as the number of germinated seeds that were inoculated/number of germinated seeds that were non-inoculated. There was a strong negative correlation between ROTS and AGERM, which was also seen in Lerch-Olson et al. (2020). Therefore, AGERM data were not used for identifying QDRL.

A linear mixed model (Bates et al., 2015) was fitted to obtain the best linear unbiased predictors (BLUPs) of each RIL for QDRL mapping using the following model: *y*_*ijk*_=μ*E*_*i*_*R*(*E*)_*ji*_*G*_*k*_*GE*_*ki*_*ε*_*ijk*_, where *y*_*ijk*_ is the observed phenotype, μ is the overall mean, *E_i* is the effect of the *i*th experiment, *R*(*E*)_*ji*_ is the effect of the *j*th replication within the *i*th experiment, *G_k* is the effect of the *k*th RIL, *GE*_*ki*_ is the interaction between the *k*th RIL and the *i*th experiment, and ɛ_*ijk*_ is the residual. All effects, except μ were treated as random. The statistical analysis was conducted using the R package lme4 (Bates et al., 2015).

### Molecular Marker Assay

To identify chromosomal regions associated with resistance to *Pythium*, all four populations were genotyped using the Illumina Infinium BARCSoySNP6K BeadChip (Song et al., 2020) at the USDA-ARS, Soybean Genomics and Improvement Lab, Beltsville, MD. Single-nucleotide polymorphisms (SNPs) in the 6k were selected from SoySNP50K (Song et al., 2013). The marker dataset was processed using GenomeStudio software (version 3.2.23). SNP markers that were monomorphic between parents of each RIL population and those which had more than 20% missing data were not used for linkage map construction. For POP4, a few SSR markers were added to two critical *Pythium* QDRL chromosomal regions. SSR markers were amplified by PCR with dye labeled forward primers (Diwan and Cregan, 1997) and analyzed by capillary electrophoresis using an Applied Biosystems 3130xl Genetic Analyzer (Foster City, CA, United States).

### Map Construction and Quantitative Disease Resistance Locus Analysis

Genetic maps for all four RIL populations were constructed using JoinMap 4.0 (van Ooijen, 2006) based on an LOD threshold of 4.0 and a maximum recombination frequency of 50% for the original grouping. Marker order and their positions within each linkage group were determined by using the maximum-likelihood algorithm and Kosambi mapping function; those unassigned to any linkage group were excluded.

MapQTL 5 software (van Ooijen, 2004) was used for the identification of each quantitative trait locus for reaction to each of the *Pythium* species assayed in this study. Using the RIL BLUP values, each round of QDRL analysis was performed in two stages: interval mapping (IM) to reveal critical chromosomal regions followed by more detailed QDRL mapping provided by the enhanced power of composite interval mapping (CIM) with the walking speed set to 1 cM.

In order to identify levels of LOD significance thresholds on both genome-wide and individual chromosome basis, 1000 iteration permutation tests were conducted. Calculated genome-wide LOD thresholds were used as a base line in significant QDRL justification, while chromosome specific LOD thresholds were used as the measure of minor QDRL consideration. In St. Clair (2010), QDRL explaining 20% or more phenotypic variation were considered to be major or large effect, and those less than 20% were considered minor QDRL. The same designation was used here.

## Results

### Phenotypic Data

The seed plate assay measured two disease reaction traits, SRS and ROTS, which were used to evaluate resistance in the four RIL populations. The disease reaction frequency distribution of the four populations can be seen in Supplementary Figures 1–4.

The correlations between the two disease reaction traits within each population and species are summarized in Table 2. Overall, the traits were strongly correlated and significant at the 0.05 probability level for all species in all populations aside from a moderate correlation for *P. irregulare* in POP4 and no correlation for *P. torulosum* in POP3 (Table 2).

**TABLE 2:** Summary of Pearson correlation coefficients within all four populations for both disease reaction traits, seed rot severity (SRS), and percent of rotted seeds in inoculated plates (ROTS), for all *Pythium* species tested, *P. sylvaticum*, *P. irregulare, P. oopapillum*, and *P. torulosum*.

*Pythium* species	POP1	POP2	POP3	POP4
*P. sylvaticum*	0.95^∗^	x	0.88^∗^	0.97^∗^
*P. oopapillum*	0.89^∗^	0.93^∗^	x	x
*P. irregulare*	x	x	0.95^∗^	0.41^∗^
*P. torulosum*	x	0.78^∗^	–	x

*^∗^Significant at the 0.05 probability level. x indicates that this species was not tested in this population. – indicates no correlation detected for this species within this population.*

### Map Construction

The constructed genetic map was verified against the soybean genome sequence version Wm82.a2. The total number of mapped markers was 2648, 2542, 2692, and 2615 in each of the populations 1, 2, 3, and 4, respectively. A total of 2542 and 2615 out of 6000 total SNP markers formed 21 linkage groups in POP2 and POP4, respectively. In POP1, 2604 SNP loci were distributed among 22 linkage groups, and in POP3, 2692 loci were distributed among 20 linkage groups. The high-density maps were subsequently used to identify chromosomal regions associated with resistance to various *Pythium* species. The number of markers mapped per chromosome for each population, the density of each map, and the genetic length of each chromosome can be seen in Supplementary Table 1.

### Mapping of Quantitative Disease Trait Loci

In POP1, which was screened for resistance to two *Pythium* spp., two minor putative QDRL were identified. One QDRL for *P. sylvaticum* was mapped for seed rot severity (SRS) and ROTS (percent rotted seed in inoculated plates), on chromosome 18 and explained 12.0 and 10.9% of the phenotypic variation, respectively (Table 3). A minor QDRL was also mapped in the same population toward *P. oopapillum* and was associated with SRS and ROTS on chromosome 2 and explained 8.9 and 10.7% of the phenotypic variation, respectively (Table 3).

**TABLE 3:** Quantitative disease resistance loci detected using composite interval mapping for *Pythium sylvaticum* and *Pythium oopapillum* in POP1 and for *Pythium torulosum* and *Pythium oopapillum* in POP2.

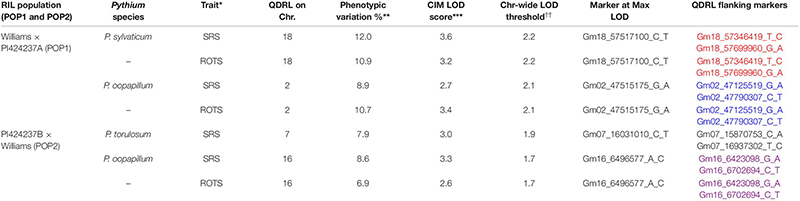

Common QDRL positions within the same population for multiple traits and/or species are shown in the same color. *SRS, seed rot severity; ROTS, percent rotted seeds in inoculated plates. **Phenotypic variation explained by the individual QDRL. ***LOD score calculated by composite interval mapping. ^††^Chromosome-wide threshold for LOD.

In POP2, two minor putative QDRL were detected, one for *P. torulosum* and another for *P. oopapillum*. The QDRL for *P. torulosum* was associated with SRS only, while the one for *P. oopapillum* was associated with SRS and ROTS. The QDRL associated with *P. oopapillum* mapped to chromosome 16 and explained 8.6 and 6.9% of the phenotypic variation, respectively (Table 3).

**FIGURE 1:**
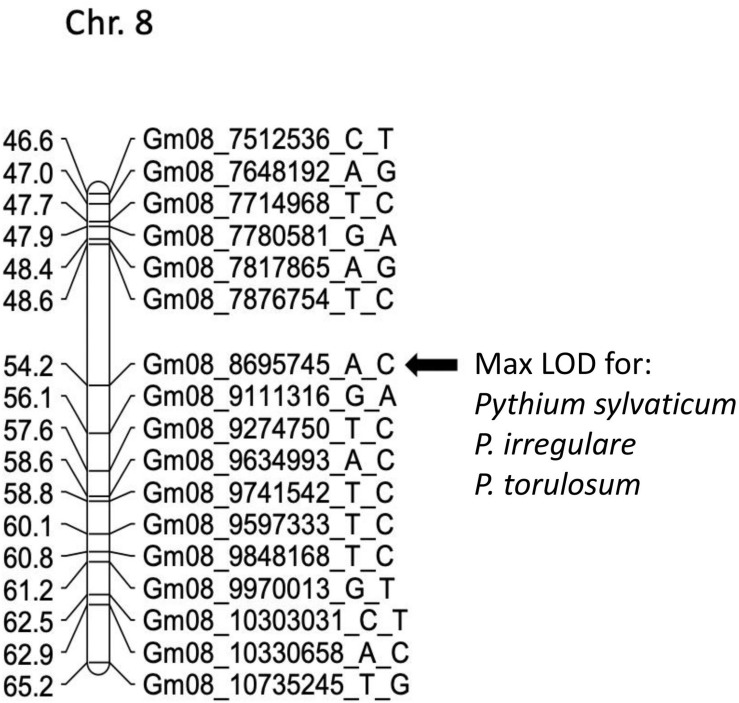
Schematic representation of a regional linkage map of chromosome 8 for POP3 with the map position for the QDRL for resistance to *Pythium sylvaticum*, *P. irregulare*, and *P. torulosum* highlighted. The SNP markers are identified on the right side and cM distances are on the left side. The closest SNP marker to the QDRL is shown by an arrow.

In POP3, a large effect QDRL was associated with the disease response traits of ROTS to *P. sylvaticum* located on chromosome 8 and flanked by SNP markers Gm08_7876754_T_C and Gm08_9111316_G_A. The CIM-based LOD score was 15.8 accounting for 21.4% of the phenotypic variation (Table 4 and Supplementary Figure 5). A regional map showing the location of this QDRL on chromosome 8 can be seen in Figure 1. The location of a minor QDRL for the SRS trait on chromosome 8 was in the same region as the large effect QDRL of the ROTS trait. A minor QDRL was also identified on chromosome 1 associated with the ROTS disease reaction trait with a corresponding CIM-based LOD score of 3.9, which explained 5.7% of the phenotypic variation. This minor QDRL on chromosome 1 was flanked by the SNP markers Gm01_51890126_A_G and Gm01_52386309_T_C (Table 4).

**TABLE 4:** Quantitative disease resistance loci detected using composite interval mapping in POP3 for *Pythium sylvaticum*, *Pythium torulosum*, and *Pythium irregulare*.

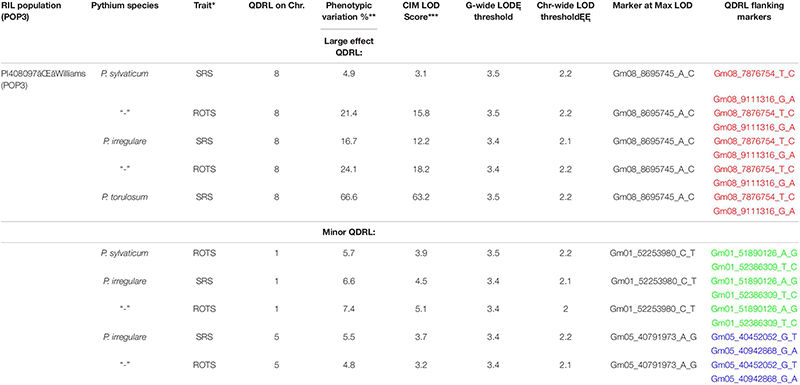

Common QDRL positions within the same population for multiple traits and/or species are shown in the same color. *SRS, seed rot severity; ROTS, percent rotted seeds in inoculated plates. **Phenotypic variation explained by the individual QDRL. ***LOD score calculated by composite interval mapping. ^†^Genome-wide threshold for LOD. ^††^Chromosome-wide threshold for LOD.

Three QDRL on chromosomes 1, 5, and 8 were identified in POP3 toward *P. irregulare* (Table 4). The QDRL on chromosome 1 appears to be the same as the QDRL identified for *P. sylvaticum* in this population (marker region shown in green in Table 4, last column). The two traits of SRS and ROTS had CIM-based LOD scores above the corresponding genome-wide LOD thresholds on chromosome 1. The CIM-based LOD values were 4.5 and 5.1 and explained 6.6 and 7.4% of the phenotypic variation, respectively (Table 4). One minor QDRL on chromosome 5 was supported by both of the measured disease traits as well. Maximum CIM-based LOD scores for this QDRL were observed between SNP markers Gm05_40452052_G_T andGm05_40942868_G_A. Corresponding explained phenotypic variation values were: 5.5 and 4.8%, respectively (Table 4). Moreover, a large effect QDRL on chromosome 8 (Supplementary Figure 6) was also identified by *P. irregulare* and was flanked by the same SNP markers as the large effect QDRL identified in the *P. sylvaticum* experiment (Gm08_7876754_T_C and Gm08_9111316_G_A). The disease reaction trait of ROTS consistently appears to be controlled by one large effect QDRL with a CIM-based LOD score of 18.2 and phenotypic variation of 24.1%. The location of a minor QDRL for the SRS trait on chromosome 8 was in the same region as the large effect QDRL of the ROTS trait explaining 16.7% of the variation.

In POP3, inoculation with *P. torulosum* resulted in the identification of a large effect QDRL on chromosome 8 for the SRS disease reaction trait only (Table 4 and Supplementary Figure 7). Position-wise this QDRL coincides with the large effect QDRL identified on chromosome 8 for both disease reaction traits in POP3 for both the *P. sylvaticum* and *P. irregulare* screening experiments (Figure 1). The CIM-based LOD score for *P. torulosum* was 63.2 and explained 66.6% of the phenotypic variation (marker region identified by the three species is shown in red in Table 4, last column).

**FIGURE 2:**
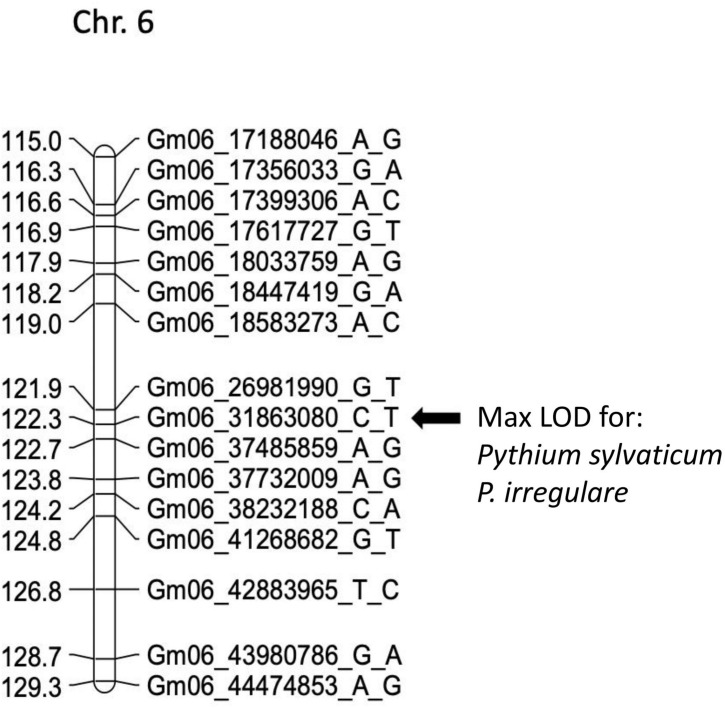
Schematic representation of a regional linkage map of chromosome 6 for POP4 with the map position for the QDRL for resistance to *Pythium sylvaticum* and *P. irregulare* highlighted. The SNP markers are identified on the right side and cM distances are on the left side. The closest SNP marker to the QDRL is shown by an arrow.

In POP4, two large effect QDRL were identified, one for *P. sylvaticum* and one for *P. irregulare* (Table 5). *P. sylvaticum* QDRL was located on chromosome 6 based on analysis of both disease-related traits (Table 5 and Supplementary Figure 8). For these traits, CIM-calculated LOD scores were 12.5 and 12.1 and the corresponding explained phenotypic variation values were 26.9 and 26.2%, which provide strong support for the relevant large effect QDRL on chromosome 6. The physical map position of this QDRL on chromosome 6 was flanked by SNP markers Gm06_26981990_G_T and Gm06_37485859_A_G. Similar to *P. sylvaticum*, a large effect QDRL for *P. irregulare* was identified on chromosome 6 in POP4 (Table 5 and Supplementary Figure 9), with explained phenotypic variation of 26.6 and 6.1%. This QDRL is flanked by SNP markers Gm06_26981990_G_T and Gm06_37485859_A_G as in the above *P. sylvaticum* case (marker region shown in red in Table 5, last column). The location of this QDRL on chromosome 6 can be observed in a regional map in Figure 2 for these two *Pythium* species.

**TABLE 5:** Quantitative disease resistance loci detected using composite interval mapping in POP4 for *Pythium sylvaticum* and *Pythium irregulare*.

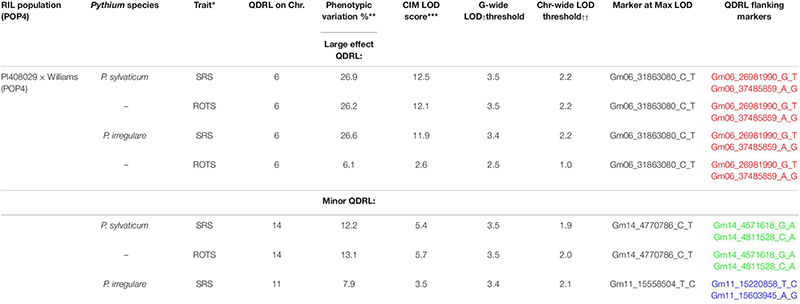

Common QDRL positions within the same population for multiple traits and/or species are shown in the same color. *SRS, seed rot severity; ROTS, percent rotted seeds in inoculated plates. **Phenotypic variation explained by the individual QDRL. ***LOD score calculated by composite interval mapping. ^†^Genome-wide threshold for LOD. ^††^Chromosome-wide threshold for LOD.

Another QDRL for *P. sylvaticum* was detected on chromosome 14 in POP4 for traits SRS and ROTS (Table 5). The CIM-calculated LOD scores were 5.4 and 5.7 with the respective contributions to phenotypic variations of 12.2 and 13.1%. The physical map position of this minor QDRL on chromosome 14 is flanked by SNP markers Gm14_4571618_G_A and Gm14_4811528_C_A (Table 5). One minor QDRL for *P. irregulare* was also detected on chromosome 11 based on the SRS disease reaction trait flanked by SNP markers Gm11_15220858_T_C and Gm11_15603945_A_G (Table 5).

## Discussion

Several studies have measured resistance toward different *Pythium* species (Rod et al., 2018; Lerch-Olson et al., 2020) and have identified QDRL, mostly minor, in RIL populations of soybean (Ellis et al., 2013; Stasko et al., 2016; Urrea et al., 2017; Klepadlo et al., 2019; Lin et al., 2018, 2020; Scott et al., 2019). Almost all of the studies have shown partial resistance aside from one study by Rosso et al. (2008) showing monogenic resistance. Our studies report multiple QDRL controlling *Pythium* resistance supporting the previously published work and contributing further data in this area.

In the current study, we used four species of *Pythium* to screen for disease resistance in four advanced generation RIL populations using multiple species per population. This is the first study to search for QDRL by screening for disease reaction to two of the *Pythium* species: *P. torulosum* and *P. oopapillum*. Two different disease reaction traits were measured in a seed root rot disease assay: SRS and the percent rotted seeds in inoculated plates (ROTS). Two large effect QDRL on chromosomes 6 and 8 and eight minor QDRL were identified among the populations to all species tested. Interestingly, a large effect QDRL was identified on chromosome 8 in POP3 conferring resistance to three species: *P. irregulare*, *P. sylvaticum*, and *P. torulosum* and another large effect QDRL on chromosome 6 in POP4 was identified for resistance to both *P. sylvaticum* and *P. irregulare*.

### Pythium irregulare

The QDRL for *P. irregulare* in POP3 on chromosome 1 was in the same chromosomal region as a QDRL found for the same species by Scott et al. (2019) in the NAM population of IA3023 × LG00-3372. The QDRL in the NAM population was found for the adjusted root weight trait and explained 6.9% of the variation. Our QDRL for this species on chromosome 1 explained up to 7.4% of the phenotypic variation. The nearest marker to this QDRL is Gm01_52253980_C_T (Table 4). Similarly, in the NAM population of IA3023 × LD02-9050 by Scott et al. (2019), a QDRL for *P. irregulare* on chromosome 5 was in a similar chromosomal location to a QDRL identified in our POP3. Their QDRL was for adjusted root weight and explained 12.2% and the marker at the peak was Gm05_389226_T_C. Our QDRL for *P. irregulare* on chromosome 5 was located near Gm05_40791973_A_G and explained up to 5.5% of the phenotypic variation. Our data combined with the findings of Scott et al. (2019) for the QDRL identified on chromosomes 1 and 5 help confirm these results. A large effect QDRL for *P. irregulare* in POP3 was found on chromosome 8 for both disease reaction traits explaining up to 24.1% of the variation (Table 4), resistance was contributed by PI 408097. Another large effect QDRL toward *P. irregulare* was detected in POP4 on chromosome 6 explaining up to 26.6% of the observed variation (Table 5) with PI 408029 contributing resistance.

### Pythium sylvaticum

*Pythium sylvaticum* was the most prevalent species detected across the midwestern United States on diseased soybean seedlings (Broders et al., 2007; Wei et al., 2010; Jiang et al., 2012; Rojas et al., 2017). We used this species to test for resistance in three of our four populations (POP1, POP3, and POP4). Multiple QDRL were found on chromosomes 1, 6, 8, 14, and 18. A large effect QDRL on chromosome 8 was identified by both disease reaction traits in POP3 (Table 4) and was contributed by PI 408097. Another large effect QDRL by both disease reaction traits was identified in POP4 on chromosome 6 (Table 5) and was contributed by PI 408029. All traits assayed for *P. sylvaticum* and *P. irregulare* detected a large effect QDRL on chromosome 6 that was flanked by the same SNP markers Gm06_26981990_G_T and Gm06_37485859_A_G by both species (Table 5). Identifying QDRL for resistance to *P. sylvaticum* is an important contribution to managing seedling disease throughout the major soybean growing areas.

### Pythium oopapillum

We evaluated resistance to *P. oopapillum*, in two populations, POP1 and POP2. Only minor effect QDRL were identified in both of these populations for reaction to this species. These are the first QDRL to be identified for *P. oopapillum* in soybean. The soybean seedling disease survey of Rojas et al. (2017) in the Midwest in 2011 and 2012 reported that *P. oopapillum* was associated with diseased soybean seedlings collected in many states, so these findings could prove useful for future breeding endeavors.

### Pythium torulosum

POP2 and POP3 were also screened for disease caused by *P. torulosum*, another prevalent species of *Pythium* in the Midwest (Dorrance et al., 2004; Jiang et al., 2012; Rojas et al., 2017; Kulkarni et al., 2018; Navi et al., 2019) that has not been subject of previous mapping studies. In POP2, only minor effect QDRL were identified; however, in POP3, a large effect QDRL was detected for the disease reaction trait, SRS, that mapped to chromosome 8 between markers Gm08_7876754_T_Cand Gm08_9111316_G_A (Table 4). Intriguingly, this QDRL is in the same chromosomal location as the large effect QDRL detected in POP3 by all four disease traits after screening by *P. sylvaticum* and *P. irregulare*. All traits for all three species assayed in this population detected a large effect QDRL on chromosome 8 that was contributed by PI 408097, and this QDRL was flanked by SNP markers Gm08_7876754_T_Cand Gm08_9111316_G_Afor all three *Pythium* species (Table 4). The implications of having a large effect QDRL in the same location on chromosome 8 for three *Pythium* species, that are some of the most prevalent in the soybean growing regions of the United States, are significant. This genomic region could be quite beneficial in breeding for partial resistance.

## Conclusion

In summary, four advanced generation RIL populations developed from new sources of resistance to *Pythium* were screened for disease reaction and subsequent mapping of QDRL toward four *Pythium* species. This is the first report of QDRL identified for two of the species, *P. oopapillum* and *P. torulosum* and the second report of QDRL for *P. sylvaticum.* The first report was by Lin et al. (2020) who identified two minor QTL on chromosomes 10 and 18 and a large effect QTL on chromosome 20 toward *P. sylvaticum.* These three *Pythium* species were among the most prevalent species recovered from diseased soybean seedlings collected in surveys of the north central soybean growing region (Dorrance et al., 2004; Broders et al., 2007; Jiang et al., 2012; Rojas et al., 2017; Navi et al., 2019). We used two disease reaction phenotypes based on a seed rot assay in which the seeds are directly infected by the pathogen to identify the QDRL. These disease reaction phenotypes measured seed rot, germination, and rotted seeds, which are related to pre-emergence damping off. Large effect QDRL were observed on chromosomes 6 and 8 in POP3 and POP4, while only minor QDRL were identified in POP1 and POP2. Of particular interest, was a large effect QDRL identified on chromosome 8 for *P. torulosum* in the same chromosomal region in POP3 to *P. sylvaticum* and *P. irregulare*. Since multiple species of *Pythium* can be recovered from a single plant, which implies that soybean seedling disease is caused by a complex of pathogens, the identification of one QDRL associated with resistance to all three *Pythium* species has major implications for breeders. Similarly, a single QDRL in the same region on chromosome 6 of POP4 was associated with reduced disease caused by *P. sylvaticum* and *P. irregulare*. In conclusion, there appears to be multiple QDRL for different species of *Pythium* that could be integrated into existing elite lines of soybean to provide partial resistance to *Pythium* species.

## Data Availability Statement

The raw data supporting the conclusions of this article will be made available by the authors, without undue reservation.

## Author Contributions

MS, AR, and AD designed the experiment. EL-O performed disease screenings. CQ and QS conducted marker assays. RB, EC, and HY analyzed the data. EC and RB wrote the initial draft. All authors were involved in reviewing, editing, and approving of the final manuscript.

## Conflict of Interest

The authors declare that the research was conducted in the absence of any commercial or financial relationships that could be construed as a potential conflict of interest.

## Supplementary Material

Supplementary Figure 1 Frequency distributions of the best linear unbiased predictor (BLUP) values in POP1 for the disease reaction traits of SRS and ROTS for *Pythium sylvaticum* **(A,B)** and *Pythium oopapillum* **(C,D)**.

Supplementary Figure 2 Frequency distributions of the best linear unbiased predictor (BLUP) values in POP2 for the disease reaction traits of SRS and ROTS for *Pythium oopapillum* **(A,B)** and *Pythium torulosum* **(C)**.

Supplementary Figure 3 Frequency distributions of the best linear unbiased predictor (BLUP) values in POP3 for the disease reaction traits of SRS and ROTS for *Pythium sylvaticum* **(A,B)**, *Pythium irregulare* **(C,D)**, and *Pythium torulosum* **(E)**.

Supplementary Figure 4 Frequency distributions of the best linear unbiased predictor (BLUP) values in POP4 for the disease reaction traits of SRS and ROTS for *Pythium sylvaticum* **(A,B)** and *Pythium irregulare* **(C,D)**.

Supplementary Figure 5 A large effect QDRL for the two disease reaction traits of seed rot severity (SRS) and the percent of rotted seeds in inoculated plates (ROTS) on chromosome 8 detected by *Pythium sylvaticum* in POP3. The ROTS trait had a CIM LOD score of 15.8. The SRS trait had a CIM LOD score of 3.1 supporting the other disease trait. The closest QDRL marker to this large effect QDRL is Gm08_8695745_A_C.

Supplementary Figure 6 A large effect QDRL detected on chromosome 8 by both disease reaction traits of seed rot severity (SRS) and the percent of rotted seeds in inoculated plates (ROTS) with *Pythium irregulare* in POP3. The traits of SRS and ROTS had CIM LOD scores of 12.2 and 18.2. Like *P. sylvaticum*, in this same population, the closest QDRL marker to this large effect QDRL isGm08_8695745_A_C.

Supplementary Figure 7 A large effect QDRL on chromosome 8 for the disease reaction trait of seed rot severity (SRS) with a CIM LOD score of 63.2 in POP3 for *Pythium torulosum* inoculation. This QDRL was also in the same chromosomal region as *P. sylvaticum* and *P. irregulare* QDRLs in this population with the closest QDRL marker being Gm08_8695745_A_C.

Supplementary Figure 8 A large effect QDRL on chromosome 6 in POP4 detected by both disease reaction traits of seed rot severity (SRS) and the percent of rotted seeds in inoculated plates (ROTS) by *Pythium sylvaticum*. The two traits had CIM LOD scores of 12.5 and 12.1, respectively. The closest QDRL marker to this large effect QDRL is Gm06_31863080_C_T.

Supplementary Figure 9 A large effect QDRL identified in POP4 on chromosome 6 by two disease reaction traits of seed rot severity (SRS) and the percent of rotted seeds in inoculated plates (ROTS) by *Pythium irregulare*. This large effect QDRL is in the same chromosomal region as the large effect QDRL after *P. sylvaticum* inoculation in this same population. The two traits had CIM LOD scores of 11.9 and 2.6. The closest QDRL marker for this large effect QDRL is Gm06_31863080_C_T.

Supplementary Table 1 A summary of the SNP marker number per chromosome for each of the four populations, the map marker density for each population, and the total number of markers mapped for each population.
