# Supplementary figures and images for: Identification of Quantitative Disease Resistance Loci Toward Four Pythium Species in Soybean
Source: Front Plant Sci. 2021 Mar 30;12:644746. doi: 10.3389/fpls.2021.644746 (PMC8042330; doi:10.3389/fpls.2021.644746)

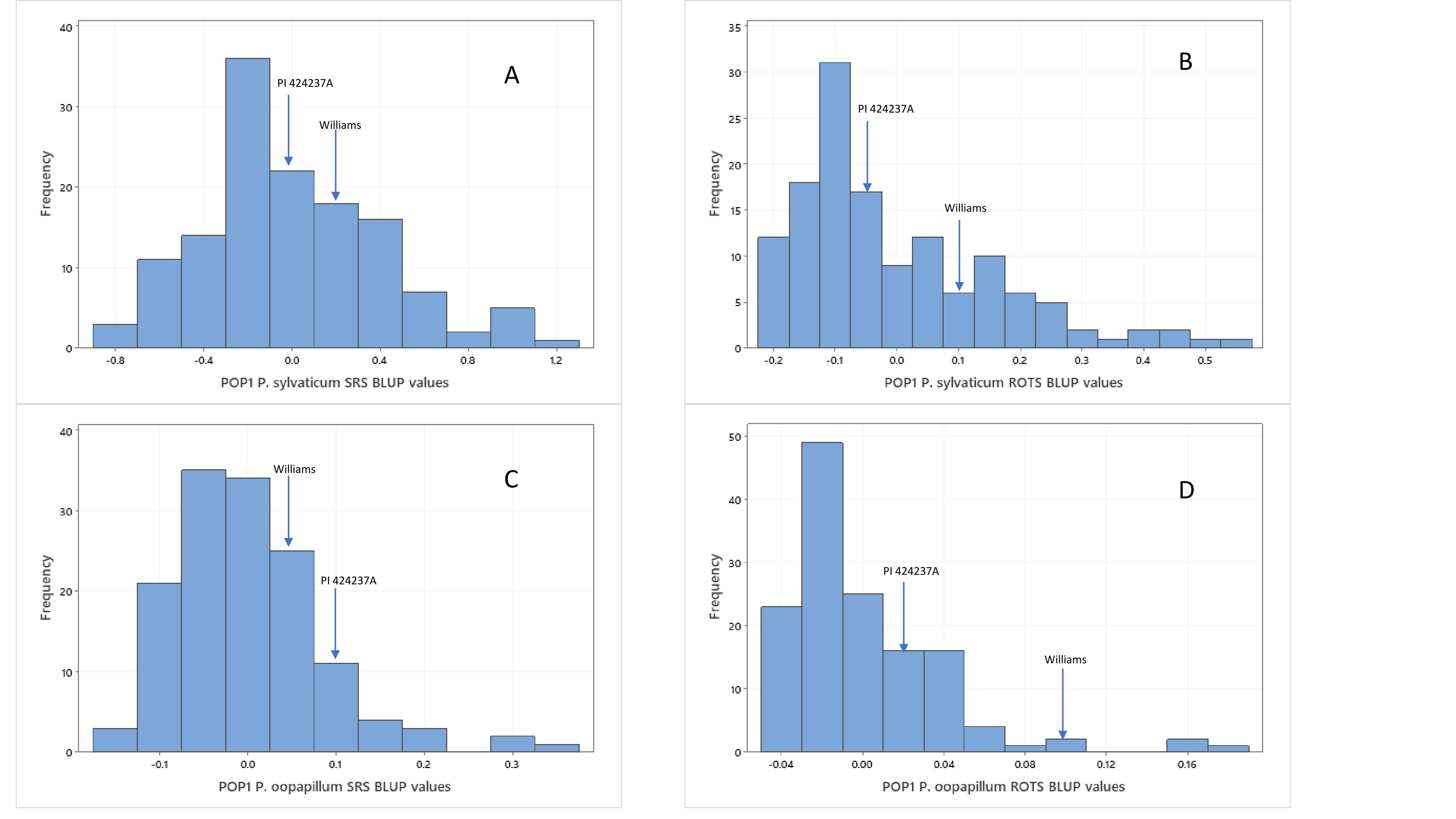

Supplement: Supplementary Figure 1 — Frequency distributions of the best linear unbiased predictor (BLUP) values in POP1 for the disease reaction traits of SRS and ROTS for Pythium sylvaticum (A,B) and Pythium oopapillum (C,D). [file Image_2.JPEG]

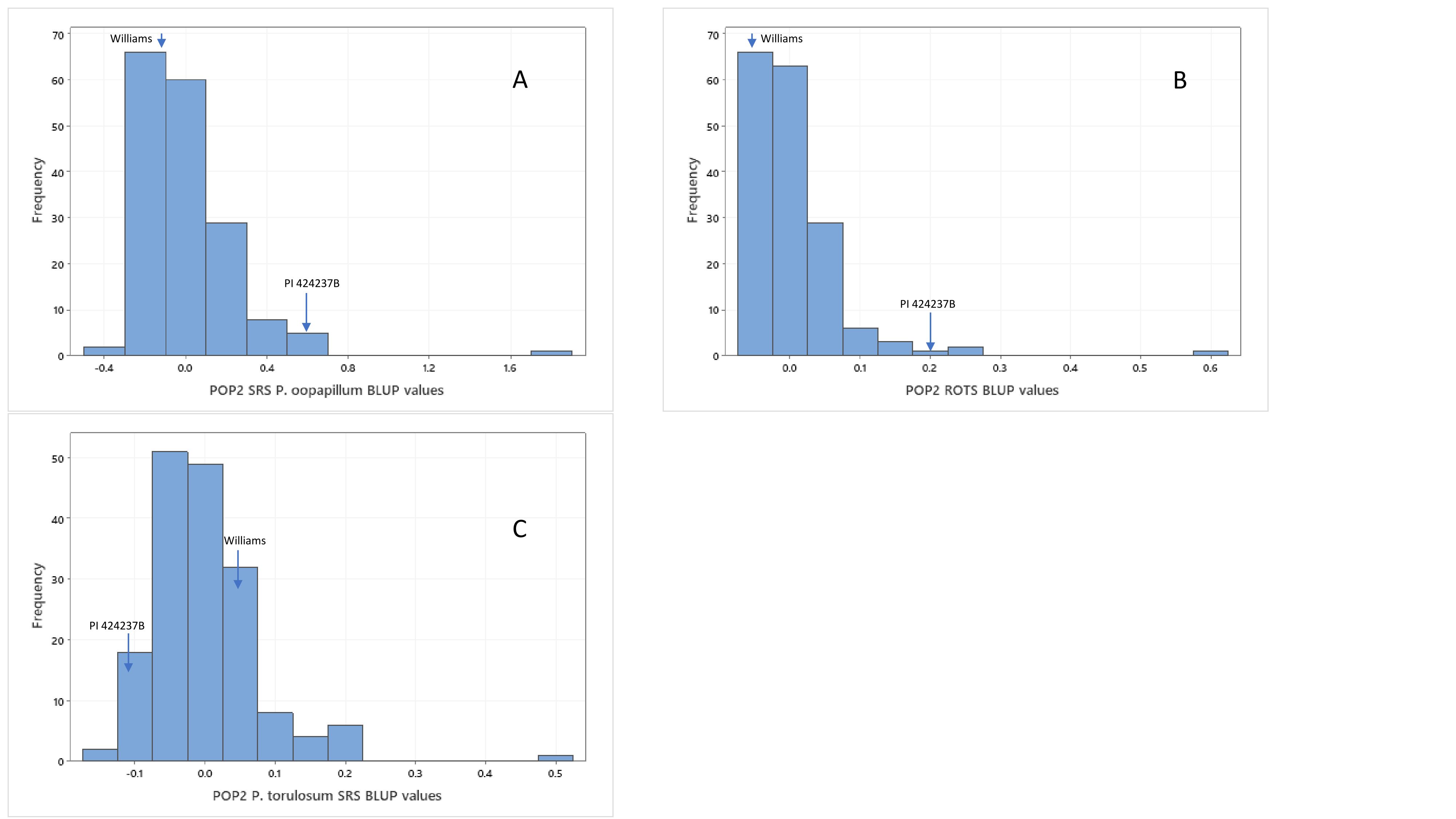

Supplement: Supplementary Figure 2 — Frequency distributions of the best linear unbiased predictor (BLUP) values in POP2 for the disease reaction traits of SRS and ROTS for Pythium oopapillum (A,B) and Pythium torulosum (C). [file Image_3.JPEG]

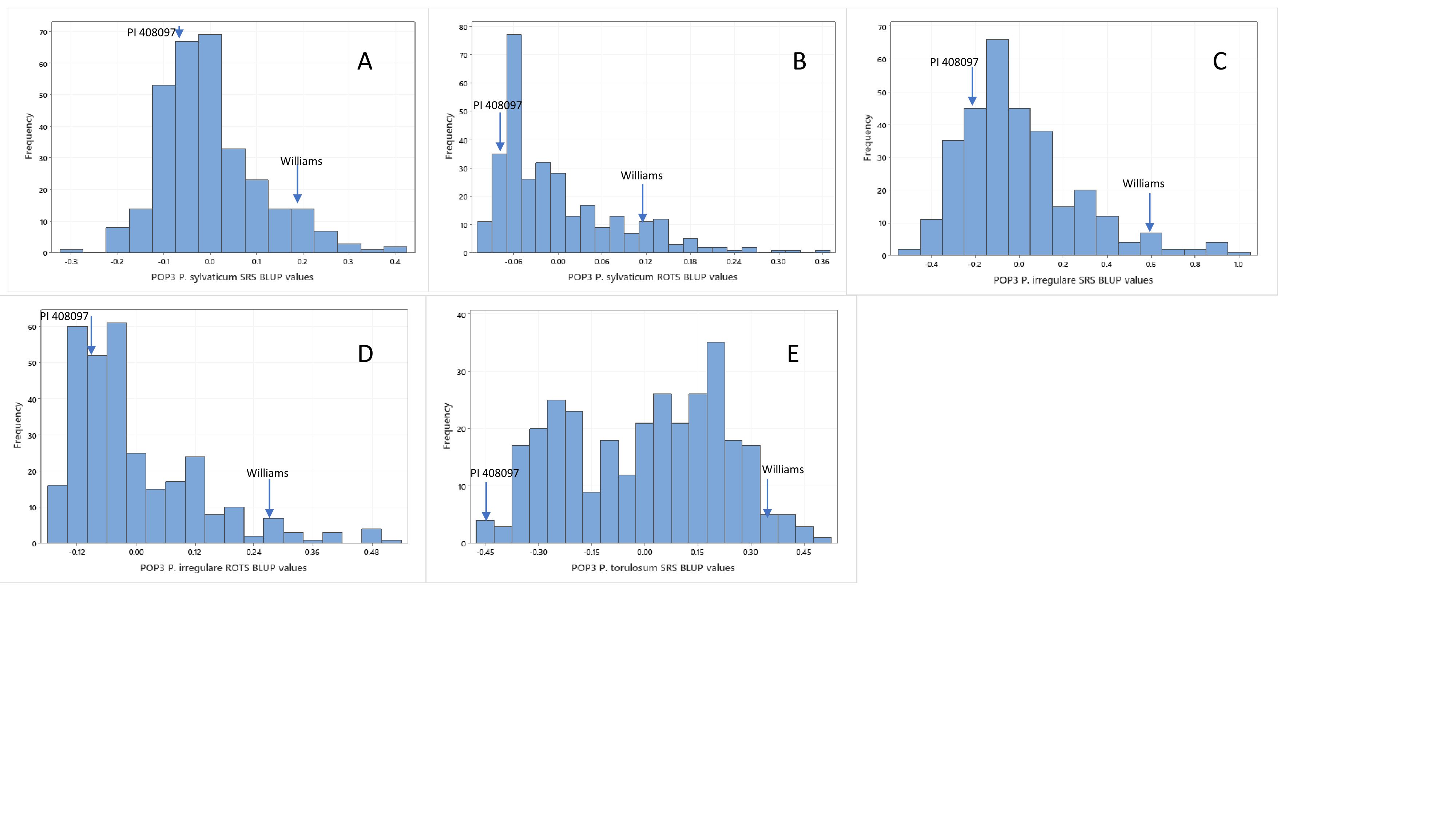

Supplement: Supplementary Figure 3 — Frequency distributions of the best linear unbiased predictor (BLUP) values in POP3 for the disease reaction traits of SRS and ROTS for Pythium sylvaticum (A,B), Pythium irregulare (C,D), and Pythium torulosum (E). [file Image_4.JPEG]

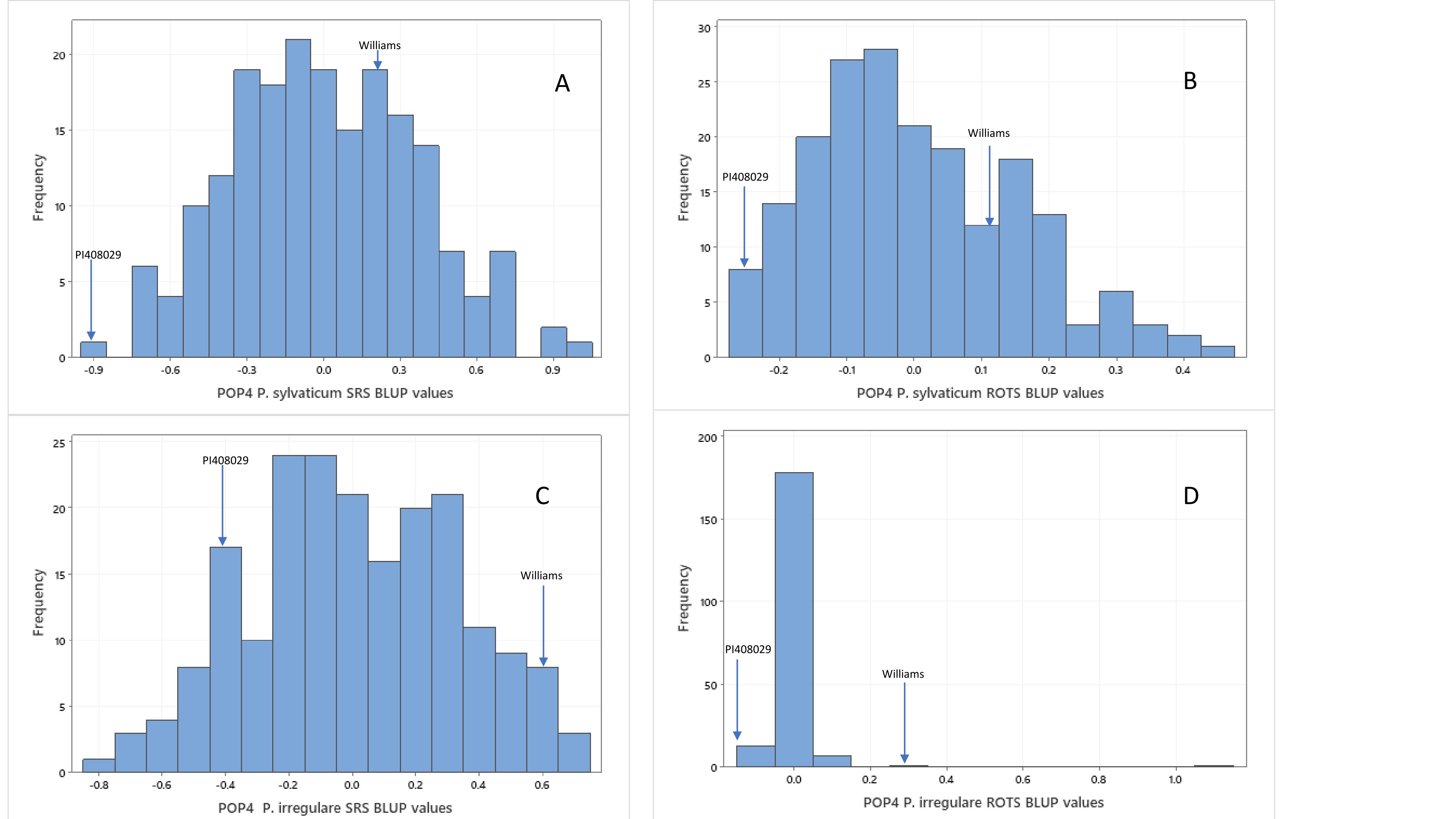

Supplement: Supplementary Figure 4 — Frequency distributions of the best linear unbiased predictor (BLUP) values in POP4 for the disease reaction traits of SRS and ROTS for Pythium sylvaticum (A,B) and Pythium irregulare (C,D). [file Image_5.JPEG]

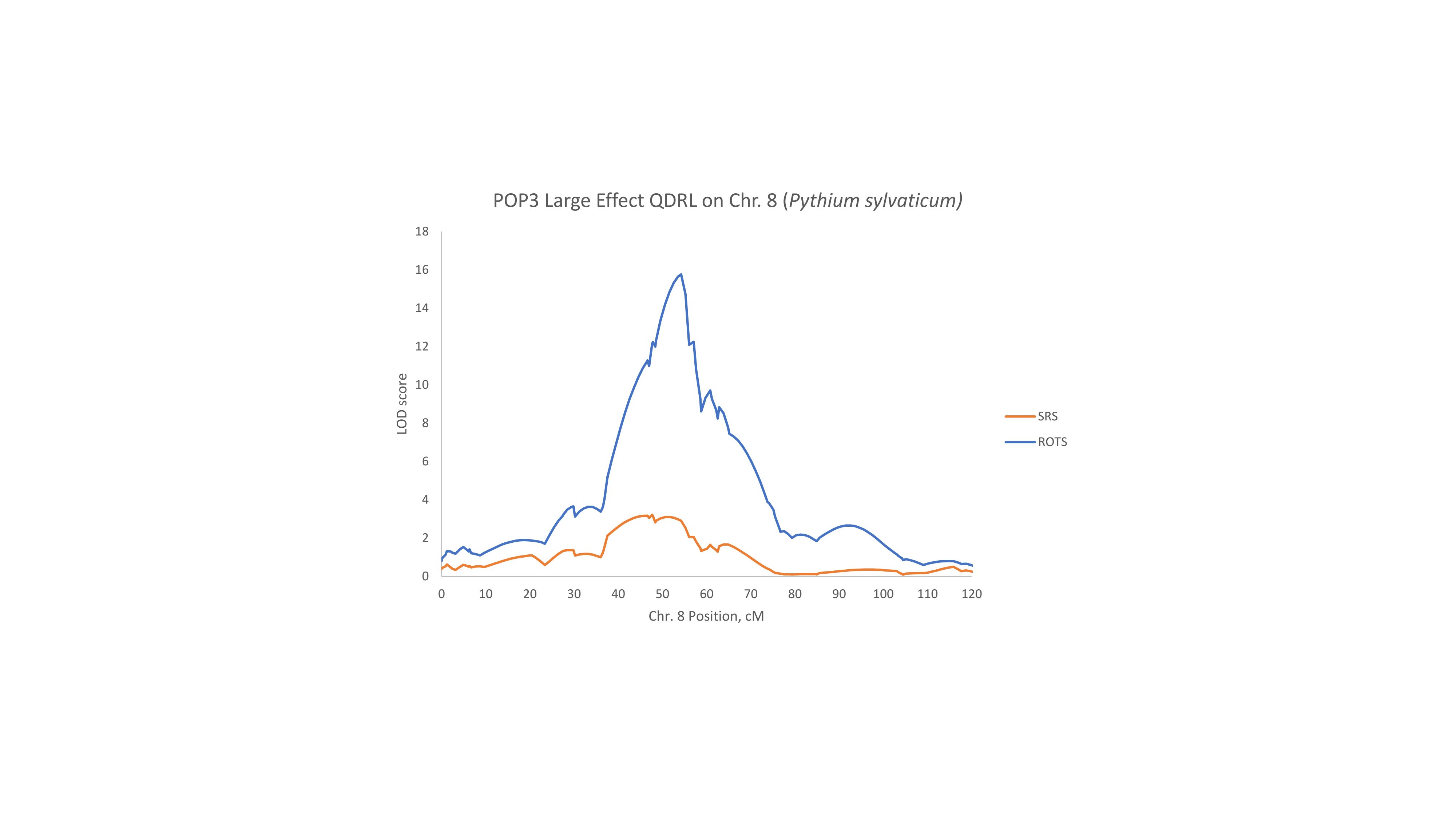

Supplement: Supplementary Figure 5 — A large effect QDRL for the two disease reaction traits of seed rot severity (SRS) and the percent of rotted seeds in inoculated plates (ROTS) on chromosome 8 detected by Pythium sylvaticum in POP3. The ROTS trait had a CIM LOD score of 15.8. The SRS trait had a CIM LOD score of 3.1 supporting the other disease trait. The closest QDRL marker to this large effect QDRL is Gm08_8695745_A_C. [file Image_6.JPEG]

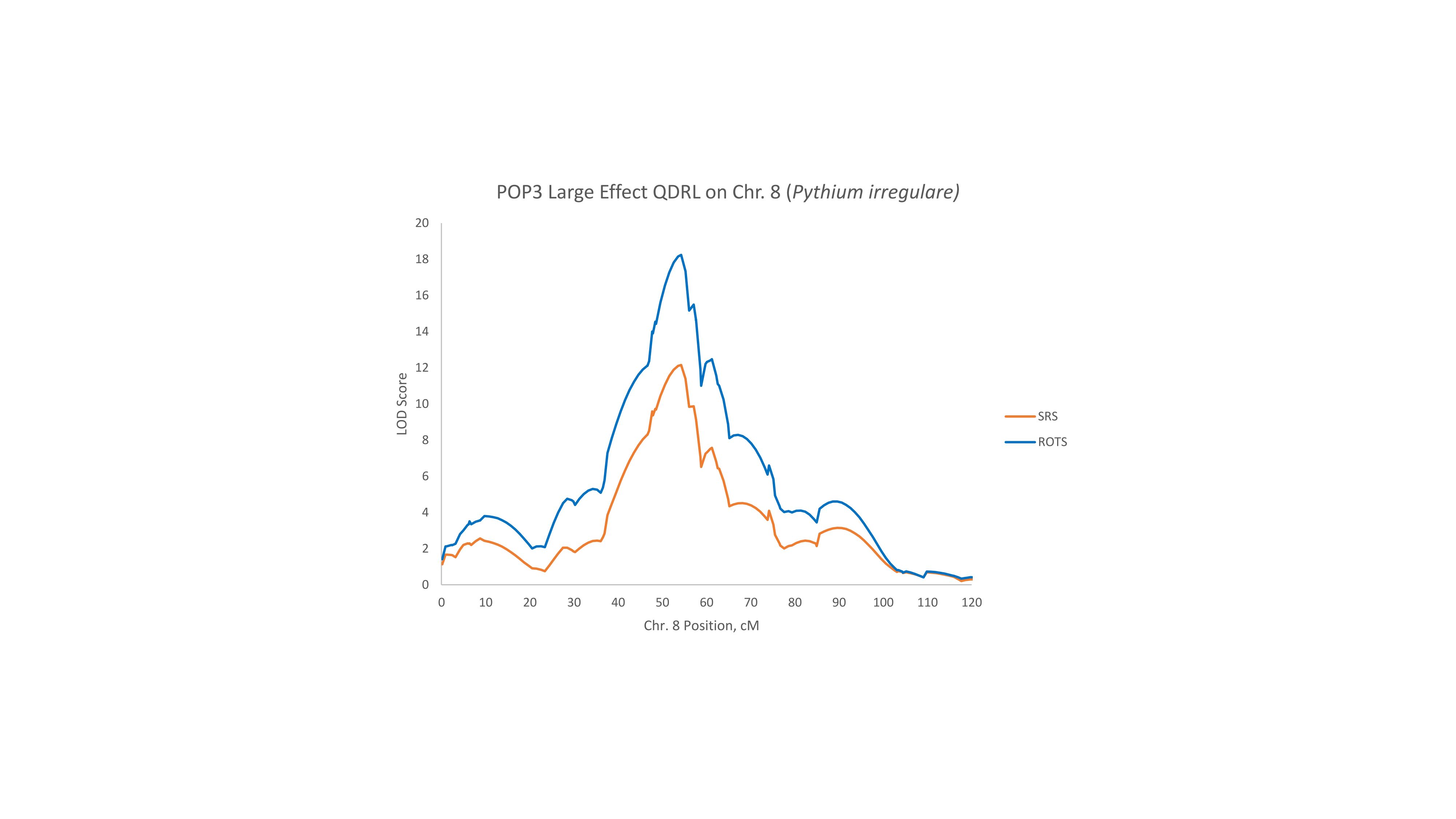

Supplement: Supplementary Figure 6 — A large effect QDRL detected on chromosome 8 by both disease reaction traits of seed rot severity (SRS) and the percent of rotted seeds in inoculated plates (ROTS) with Pythium irregulare in POP3. The traits of SRS and ROTS had CIM LOD scores of 12.2 and 18.2. Like P. sylvaticum, in this same population, the closest QDRL marker to this large effect QDRL isGm08_8695745_A_C. [file Image_7.JPEG]

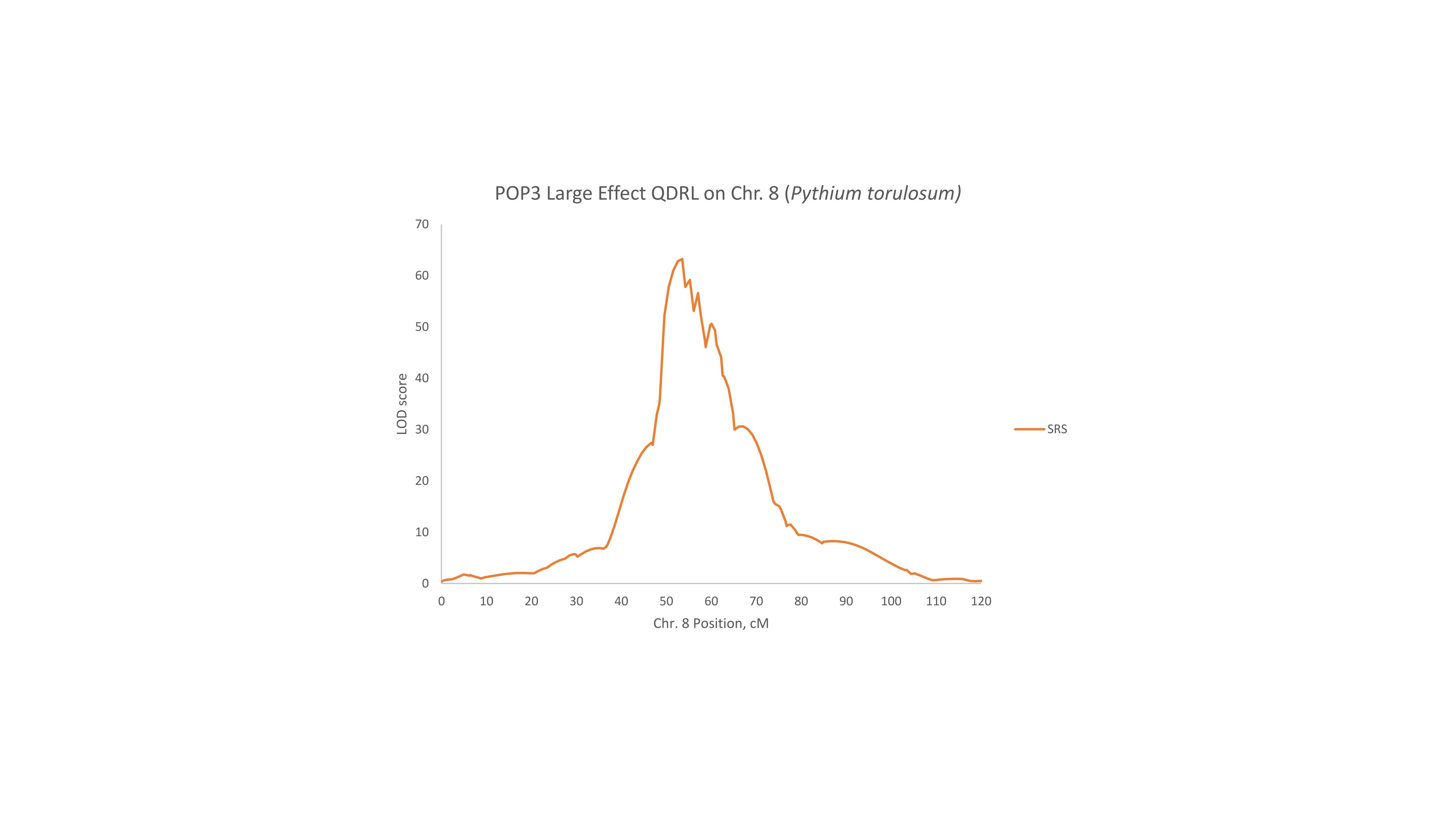

Supplement: Supplementary Figure 7 — A large effect QDRL on chromosome 8 for the disease reaction trait of seed rot severity (SRS) with a CIM LOD score of 63.2 in POP3 for Pythium torulosum inoculation. This QDRL was also in the same chromosomal region as P. sylvaticum and P. irregulare QDRLs in this population with the closest QDRL marker being Gm08_8695745_A_C. [file Image_8.JPEG]

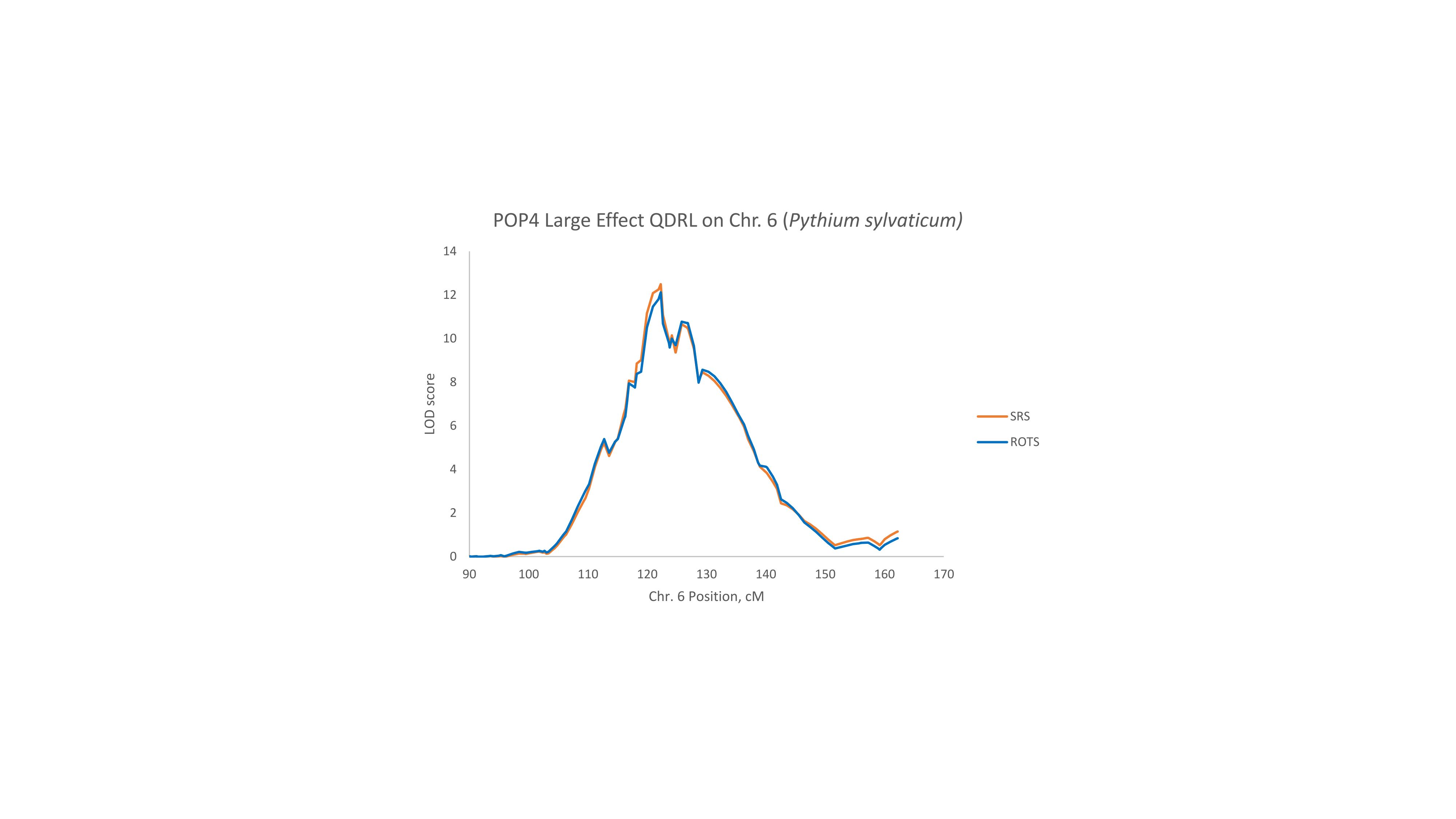

Supplement: Supplementary Figure 8 — A large effect QDRL on chromosome 6 in POP4 detected by both disease reaction traits of seed rot severity (SRS) and the percent of rotted seeds in inoculated plates (ROTS) by Pythium sylvaticum. The two traits had CIM LOD scores of 12.5 and 12.1, respectively. The closest QDRL marker to this large effect QDRL is Gm06_31863080_C_T. [file Image_9.JPEG]

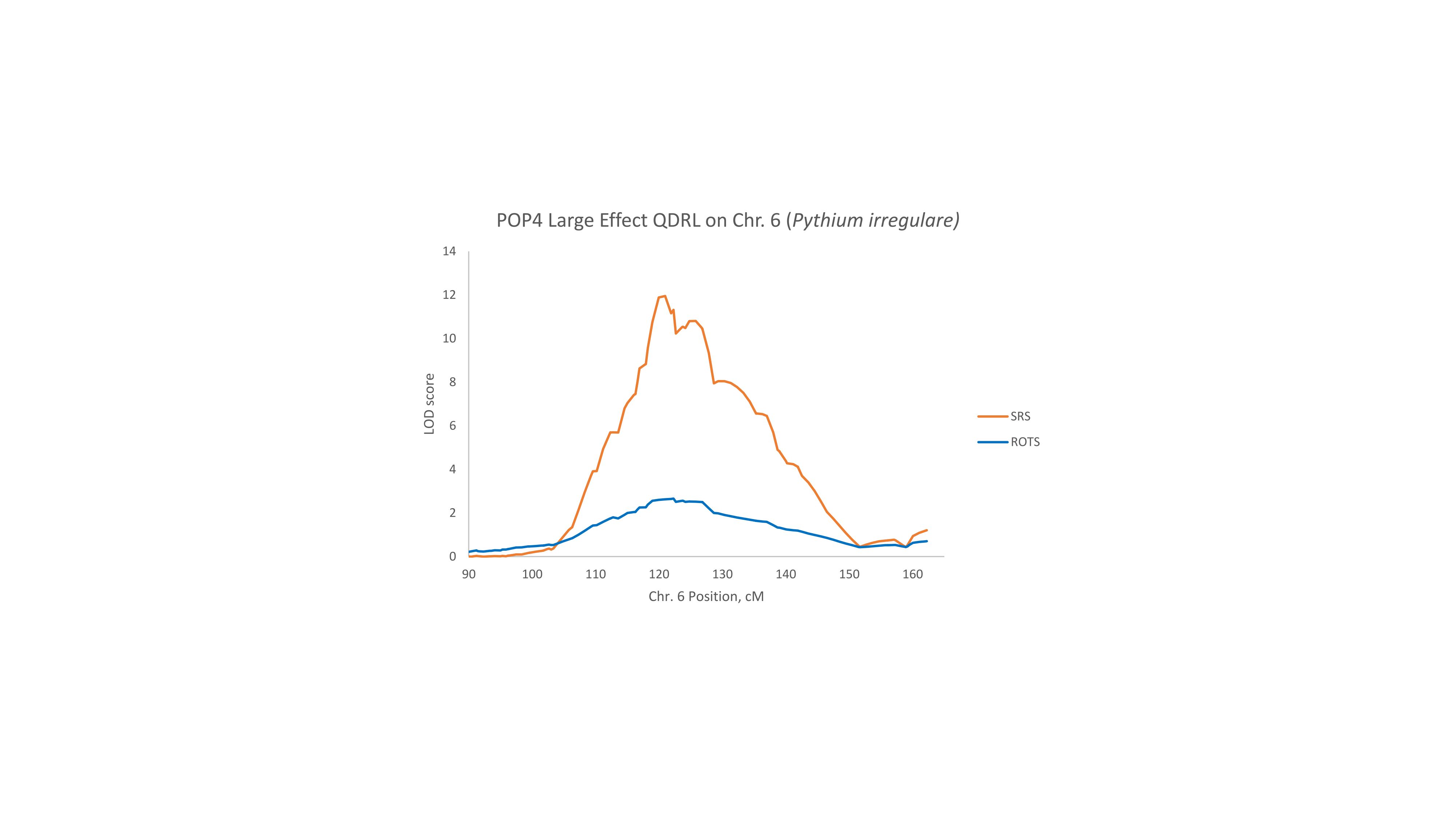

Supplement: Supplementary Figure 9 — A large effect QDRL identified in POP4 on chromosome 6 by two disease reaction traits of seed rot severity (SRS) and the percent of rotted seeds in inoculated plates (ROTS) by Pythium irregulare. This large effect QDRL is in the same chromosomal region as the large effect QDRL after P. sylvaticum inoculation in this same population. The two traits had CIM LOD scores of 11.9 and 2.6. The closest QDRL marker for this large effect QDRL is Gm06_31863080_C_T. [file Image_10.JPEG]

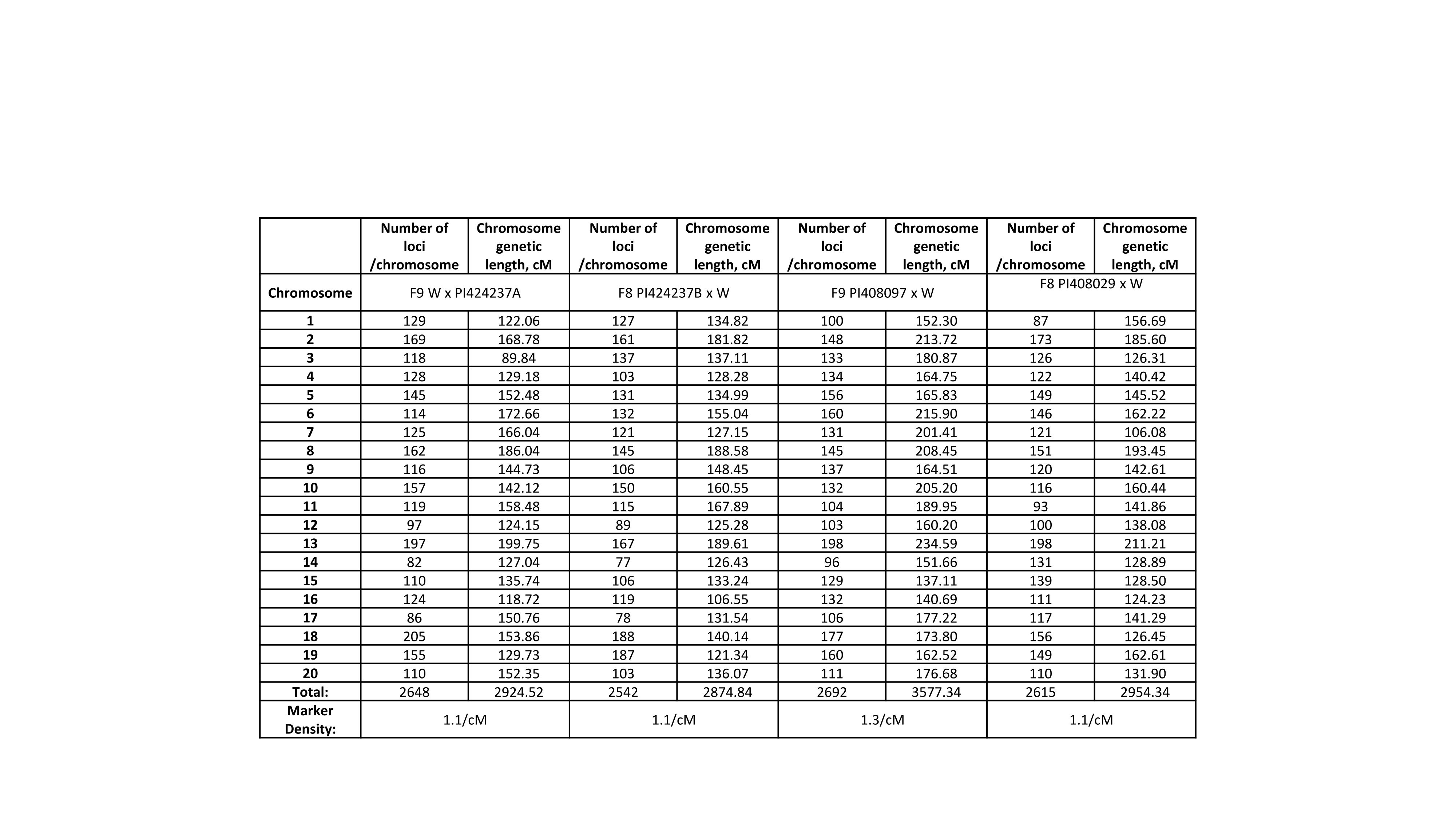

Supplement: Supplementary Table 1 — A summary of the SNP marker number per chromosome for each of the four populations, the map marker density for each population, and the total number of markers mapped for each population. [file Image_1.JPEG]
